# COVID-19 vaccination and corneal allograft rejection- a review

**DOI:** 10.3389/fcimb.2023.1307655

**Published:** 2023-12-15

**Authors:** Natalie E. Allen, Jie Zhang, Charles N. J. McGhee

**Affiliations:** Department of Ophthalmology, New Zealand National Eye Centre, Faculty of Medical and Health Sciences, University of Auckland, Auckland, New Zealand

**Keywords:** COVID-19, vaccination, cornea, keratoplasty, rejection

## Abstract

**Aim:**

To provide a comprehensive literature review on the perceived correlation between COVID-19 vaccination and corneal allograft rejection, and to characterize risk factors, time course, graft outcomes and proposed immunological basis.

**Methods:**

A literature review was conducted in August 2023 using 4 electronic databases: PubMed, EMBASE, MEDLINE and Scopus. Articles were sourced using key words associated with COVID-19 vaccination and corneal graft. All articles were screened for relevance by abstract review. Duplicates and articles related to COVID-19 infection were excluded. No time limits were set. Additional literature searches regarding cause of corneal graft rejection, rates of graft rejection associated with other vaccines and the cellular mechanism of rejection were also performed.

**Results:**

262 articles were identified from the literature search. 37 papers were included in the analysis based on defined inclusion criteria. This consisted of systematic reviews (n=6), review articles (n=5), retrospective studies (n=3), case series (n=8), letter to the editor (n=1) and case reports (n= 14). The majority of reported allograft rejections were in penetrating keratoplasties. Risk factors for COVID-19 vaccination associated rejection were previous allograft rejection episodes, repeat grafts and penetrating keratoplasty. Most reported rejection episodes were mild and resolved with treatment. Notably, several studies reported nil increase in corneal allograft rejection episodes over the COVID-19 vaccination period. Rejection episodes are associated with a broad spectrum of other vaccines and the complete pathophysiology is undetermined.

**Conclusion:**

Corneal allograft rejection appears to be a rare complication of COVID-19 vaccination most frequently observed in high-risk corneal transplants. The true extent of this correlation remains controversial; however, clinician awareness of this risk is essential to its mitigation. Patient counselling around symptom monitoring following vaccination and discussion around topical steroid prophylaxis may be prudent.

## Introduction

Severe acute respiratory syndrome coronavirus 2 (SARS-CoV-2) is a highly contagious virus that swept across the globe in December 2019 resulting in the coronavirus disease (COVID-19) pandemic (Wiersinga et al., 2020). Estimated to have resulted in excess deaths of 14.8 million globally, the pandemic was declared to no longer be a “global health emergency” by the World Health Organization (WHO) on May 6th 2023 (Msemburi et al., 2023). This was due in part to the development and distribution of recombinant mRNA vaccines, of which over 13 billion doses have been administered globally by July 2023 (Mathieu et al., 2020).

Corneal allograft transplantation is an end-stage treatment for several corneal pathologies (Kim et al., 2016; Alio et al., 2021; Yin, 2021). The terms “corneal transplantation or corneal graft” refer to several different operations involving corneal allograft tissue of varying layers and thickness. Penetrating keratoplasty (PK) involves transplantation of all five key layers i.e. from anterior to posterior, the corneal epithelium, Bowman’s layer, corneal stroma, Descemet’s membrane and endothelium. Deep anterior lamellar keratoplasty (DALK) typically involves transplantation of the corneal epithelium and full-thickness stroma without the highly immunogenic endothelium (Crawford et al., 2013; Yin, 2021).

Descemet’s stripping (automated) endothelial keratoplasty (DS(A)EK) is a lamellar procedure that utilizes donor tissue comprising a thin layer of deep corneal stroma plus the subjacent Descemet’s membrane and endothelium. Descemet’s membrane endothelial keratoplasty (DMEK) utilizes donor tissue that includes Descemet’s membrane and the endothelium.

Corneal transplantation is arguably the most successful tissue transplantation due to its (relative) immune privilege (Niederkorn, 2006; Maharana et al., 2023). It does not require HLA matching or routine systemic immunosuppression due to lymphangiogenic and hemangiogenic privilege (Hori et al., 2019; Maharana et al., 2023). The cornea has no blood vessels or lymphatics, with inhibitory collagens within the corneal stroma which inhibit blood vessel growth. The anatomy of the limbus impedes vascularization of the adjacent cornea (Bron et al., 1997). Collagen fibers I and V are integral to maintaining corneal clarity and impede the growth of vessels into the corneal stroma. The limbal arcade remains 1-5mm outside of the corneal limbus and has no smooth muscle but contains actin filaments under normal physiological conditions. Under hypoxic conditions, these actin filaments are impeded by nitric oxide released by the vascular endothelium, causing vascular dilation and subsequent neovascularization (Hori et al., 2019). The corneal epithelium also expresses several factors such as VEGF receptor3 (VEGFR-3) and TNF-related apoptosis-inducing ligand (TRAIL) which are angiostatic factors (Hori et al., 2019; Maharana et al., 2023). This immune privilege is variable between layers of the cornea, with the epithelium being less immune privileged than the endothelium. This explains the lower rates of graft rejection in DSAEKs and DMEKs (9%) compared with PKs (21%) (Zhang et al., 2019; Yin, 2021; Maharana et al., 2023).

Corneal allograft rejection describes a pattern of symptoms and clinical signs indicating that the host immune system is responding to the allograft, and is the leading cause of corneal graft failure (Panda et al., 2007). The most common indication for repeat corneal transplantation is corneal endothelial failure (Lu et al., 2019). Rejection episodes occasionally may be relatively asymptomatic in mild cases but are more typically characterized by several symptoms including photophobia, ocular redness (often circumcorneal), blurred vision, discomfort and epiphora. By definition at least one of the following clinical signs should be present; epithelial rejection line, sub-epithelial infiltrates (Krachmer spots), stromal oedema, and anterior chamber inflammation with keratic precipitates on the endothelium and/or an endothelial rejection line (Khodadoust’s line). This process typically occurs after 2 weeks or longer postoperatively in primary allograft rejection and the changes are usually limited to the graft tissue (Panda et al., 2007; Alio et al., 2021).

It is postulated that vaccines alter the immune privilege of the eye. Surgery induces expression of major histocompatibility complex class-Ii expression in the antigen presenting cells (APCs) of the cornea, namely, corneal dendritic cells (cDCs), and induces the expression of costimulatory molecules. cDCs enter a mature state. Both donor and/or host cDCs in the central cornea can migrate to the host peripheral cornea, then to the lymph nodes. T cells within lymph nodes then induce an immune response involving induction of cytotoxic T cells and proinflammatory IgG antibody production within B cells. This leads to a Delayed Type Hypersensitivity response, which then causes graft rejection. This is modulated by two active mechanisms: anterior chamber-associated immune deviation (ACAID) and the intraocular immunosuppressive microenvironment (Niederkorn, 2007; Maharana et al., 2023). ACAID is induced systemic tolerance to alloantigens within the anterior chamber. When antigens arrive in the anterior chamber, antigen-bearing antigen-presenting cells (APCs) travel via the trabecular meshwork to the spleen, which produces TGF-beta, TSP-1, and MIP-2. These factors attract natural killer T cells (NKT cells), which in turn attract marginal zone B cells (Maharana et al., 2023). These activities create an environment in which responding T cells differentiate into CD4+ T regulatory cells (Tregs), which inhibit the Delayed Type Hypersensitivity response. The immunosuppressive microenvironment involves several soluble factors within the anterior chamber and cell surface molecules within the cornea, iris and ciliary body which act to hinder the usual systemic immune response to pathogens. These include Calcitonin gene-related peptide which suppresses macrophage activity, and Vasoactive intestinal peptide which suppresses T cells (Gonzalez-Rey et al., 2007; Hori et al., 2019).

Vaccination against various infectious pathologies may compromise corneal immune privilege and is anecdotally associated with corneal allograft rejection (Steinemann et al., 1988; Streilein, 2003; Melo-González et al., 2021; Dugan and Mian, 2022). Recent case reports and retrospective case series have described links between COVID-19 vaccination and ocular pathology including corneal graft rejection (Abousy et al., 2021; Crnej et al., 2021; Al-Dwairi et al., 2022). Therefore, this literature review aims to provide an in-depth analysis of the perceived correlation between COVID-19 vaccination and corneal allograft rejection, supplemented by the characteristics, time course, clinical outcomes and consideration of the underlying immunological basis.

## Method

**Figure 1:**
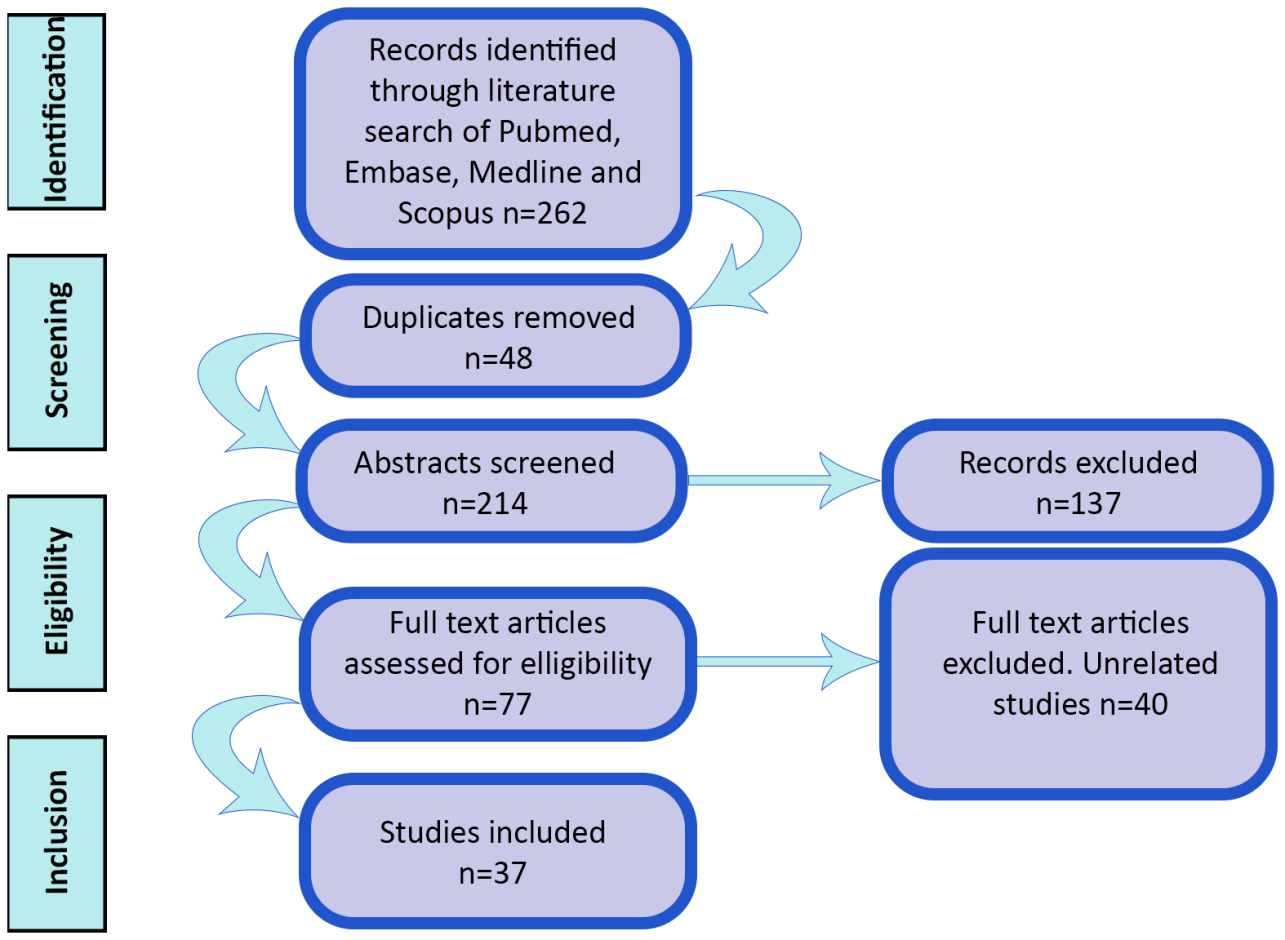
Summary of literature search.

We conducted a literature search in August 2023 using 4 electronic databases: MEDLINE, SCOPUS, PubMed and EMBASE. We performed a search of all English language literature using the following terms “Covid-19, coronavirus-19, SARS-CoV-2, 2019-nCoV, vaccine, vaccination, immunization, immunization, cornea, corneal graft, keratoplasty, corneal transplantation, corneal tissue, DSAEK, DMEK, PKP” and their synonyms, matching to MeSH headings when searching in Pubmed. No time range limits were set for the literature search. All case reports, case series, systematic reviews, narrative reviews and meta-analyses were included. Correspondence with no patient data was excluded. The references of the remaining publications were also reviewed to identify relevant literature. The full texts of all remaining studies were screened as outlined in **Figure 1**, and if they contained COVID-19 vaccination and corneal grafts, they were included. Studies of COVID-19 infection alone and corneal allograft rejection alone were excluded. Studies of other types of vaccination and corneal allograft rejection were excluded from the analysis but read for the purpose of better understanding the potential immunological mechanisms behind rejection.

## Results

Across all four databases, 262 results articles were identified. Of these, 48 were excluded as duplicates, 6 were excluded due to publication type and 170 were excluded due to relevance. This left 37 papers on COVID-19 vaccination and corneal allograft rejection in the literature. This included 6 systematic reviews, 5 review articles, 3 retrospective studies, 8 case series, 14 case reports and one letter to the editor. There were numerous different variations of COVID-19 vaccination described. Within the non-review papers mRNA-1273 (Moderna) was described 7 times, AZD1222 (Oxford-AstraZeneca) was described 10 times, BNT162b2 (Pfizer) was described 15 times, BBIBP-CorV (Sinopharm) was described 8 times, Sinovac was described once and BBV152 (Covaxin) was described once. This is summarized in **Table 1**.

**Table 1:** COVID-19 and Corneal Graft Rejection.

Age Gender	Risk factors	Vaccine (dose)	Type of corneal transplant	Interval between vaccine and rejection (days)	Ocular findings	Treatment	Graft failure/Conclusions
**63M** (**Balidis et al., 2021**)	Repeat graft	AZD122 2 (1)	DSAEK	10	Stromal oedema	Dexamethasone eye drops and hypertonic ointment every 2 h	Yes
**Approx 60M** (**Ghiasian et al., 2023**)	Not applicable (N/A)	BBIBP- CorV (1)	PK	3	Corneal oedema	Topical steroid	Yes
**50M** (**Ghiasian et al., 2023**)	Previous rejection	BBIBP- CorV (1)	PK	11	Corneal oedema and KPs	Topical steroid	Yes
**40M** (**Ghiasian et al., 2023**)	N/A	BBIBP- CorV (2)	PK	54	Corneal oedema and KPs	None	Yes
**80M** (**Ghiasian et al., 2023**)	Previous rejection	BBIBP- CorV (3)	PK	6	Corneal oedema and KPs	Topical and oral steroid	Yes
**94F** (**Forshaw et al., 2022**)	N/A	BNT162 b2 (1)	DMEK bilateral	14	Diffuse cornea oedema and DM folds	Dexamethasone/tobramycin 6x per day and hypertonic 4x per day	Yes
**51M** (**Yu et al., 2022**)	Previous rejection, glaucoma	mRNA- 1273 (1)	PK	3	Corneal oedema and KPs	Topical steroids	Yes
**64F** (**Balidis et al., 2021**)	Repeat graft	mRNA- 1273 (2)	PK	7	Diffuse corneal oedema and KPs.	Dexamethasone eye drops hourly and intracameral fortecortin injections	Yes
**63F** (**Simão and Kwitko, 2022**)	Previous rejection, previous LASIK	Sinovac	PK	1	Corneal oedema and AC inflammation	Topical dexamethasone every hour, 0.5% timolol maleate, 0.03% bimatoprost and polydimethylsiloxane 4 times a day	Yes
**69M** (**Balidis et al., 2021**)	Diabetes on insulin	AZD122 2 (1)	PK	5	Corneal oedema	Subconjunctival dexamethasone injections and combined oral (methylprednisolone) and topical (dexamethasone) corticosteroid therapy	No
**55M** (**Molero-Senosiain et al., 2022**)	N/A	AZD122 2 (1)	PK	7	Stromal oedema, DM folds, KPs	Topical steroid	No
**28F** (**Nahata et al., 2022**)	N/A	AZD122 2 (1)	FLEK	14	Khoudadoust line, DM folds, AC inflammation, stromal oedema	Topical prednisolone 1% hourly, topical homatropine eye drops three times and with oral methylprednisolone 50mg once daily for 1 week	No
**64M** (**Park et al., 2023**)	Previous rejection	AZD122 2 (1)	PK	2	Corneal oedema and KPs	Topical and systemic steroid	No
**35M** (**Parmar et al., 2021**)	Previous rejection with graft neovasculari sation	AZD122 2 (1)	PK	4	Corneal oedema, KPs, AC inflammation, pupil deformity	Topical prednisolone acetate 1% hourly, IV methylprednisolone for 3 days, switching to oral prednisone 60mg PO OD	No
**62M** (**Ravichandran and Natarajan, 2021**)	N/A	AZD122 2 (1)	PK	21	Khodadoust’s line, corneal oedema	N/A	N/A
**18M** (**Swarup and Mehta, 2023**)	Young	AZD122 2 (1)	DALK	13	Ciliary haze, stromal oedema	Topical prednisolone acetate 1% every hour, cycloplegics and lubricants	No
**61M** (**Molero-Senosiain et al., 2022**)	N/A	AZD122 2 (2)	PK	30	Corneal oedema and AC inflammation	Topical steroid and IV methylprednisolone	No
**79M** (**Rajagopal R. and Priyanka T.M., 2022**)	Previous rejection	AZD122 2 (2)	PK	42	Stromal oedema	Hourly topical steroids and oral steroids	No
**36F** (**Mohammadzadeh M. et al., 2022**)	N/A	BBIBP- CorV (1)	PK	2	Corneal oedema	Topical betamethasone	No
**54F** (**Mohammadzadeh M. et al., 2022**)	N/A	BBIBP- CorV (1)	PK	7	Corneal oedema	Topical betamethasone	No
**40M** (**Ghiasian et al., 2023**)	N/A	BBIBP- CorV (2)	PK	65	Corneal oedema and KPs	Topical steroid	No
**20M** (**Ghiasian et al., 2023**)	N/A	BBIBP- CorV (2)	PK	117	Corneal oedema and KPs	None	No
**15F** (**Swarup and Mehta, 2023**)	Young, Neovascular isation	BBV152 (1)	DALK	9	Stromal oedema	Topical prednisolone acetate 1% every hour, cycloplegics and lubricants	No
**71M** (**Crnej et al., 2021**)	Smoking	BNT162 b2 (1)	DMEK	7	Diffuse corneal oedema	Topical dexamethasone every 2 h	No
**40M** (**Eduarda Andrade E Andrade M. et al., 2022**)	N/A	BNT162 b2 (1)	PK	10	Subepithelial bullae, Khodadoust line and KPs	Oral prednisone 20mg, hourly topical prednisolone acetate and subconjunctival dexamethasone	No
**15M** (**Marziali et al., 2022**)	Young age	BNT162 b2 (1)	PK	12	Conjunctival injection, DM folds, corneal oedema	Topical dexamethasone sodium phosphate 2% 6x per day	No
**72F** (**Molero-Senosiain et al., 2022**)	Previous rejection	BNT162 b2 (1)	DSAEK	14	Diffuse corneal oedema	Topical dexamethasone	No
**82F** (**Molero-Senosiain et al., 2022**)	N/A	BNT162 b2 (1)	DSAEK	14	Corneal oedema	Topical steroid	No
**48F** (**Molero-Senosiain et al., 2022**)	Previous rejection	BNT162 b2 (1)	PK	30	Microcystic oedema and DM folds	Topical dexamethasone	No
**44F** (**Nioi M. et al., 2021**)	N/A	BNT162 b2 (1)	PK	13	Diffuse corneal oedema, KPs, DM folds, AC inflammation	Topical dexamethasone and Vitamin D	No
**66F** (**Phylactou et al., 2021**)	HIV (well controlled)	BNT162 b2 (1)	DMEK	7	Diffuse corneal oedema, KPs, AC inflammation	Topical dexamethasone	No
**68F** (**Rallis et al., 2022**)	Previous rejection	BNT162 b2 (1)	PK	4	Diffuse PEEs, DM folds, corneal oedema and AC inflammation	Topical dexamethasone and oral acyclovir	No
**72M** (**Wasser et al., 2021**)	Previous rejection	BNT162 b2 (1)	PK	13	Ciliary injection, DM folds, corneal oedema and KPs	Oral and topical steroid	No
**56M** (**Wasser et al., 2021**)	Previous rejection	BNT162 b2 (1)	PK	12	Diffuse corneal oedema, KPs and AC inflammation	Oral prednisone 60mg daily and topical dexamethasone hourly	No
**73F** (**Abousy et al., 2021**)	N/A	BNT162 b2 (2)	DSAEK Bilateral	4	Thickened corneas with DF	Prednisolone acetate 1% eye drops four times daily	No
**73F** (**Abousy et al., 2021**)	N/A	BNT162 b2 (2)	DSAEK Bilateral	9	Moderate conjunctival congestion, diffuse corneal edema, KPs, and AC inflammation	Prednisolone acetate 1% eye drops four times daily	No
**72M** (**Gouvea et al., 2022**)	N/A	BNT162 b2 (2)	KLAL and PK	30	Perilimbal engorgement and tortuosity of vessels	Hourly difluprednate 0.05% drops, tacrolimus 0.1% ointment before bed, and his oral tacrolimus was increased to 2 mg twice daily	No
**83F** (**Phylactou et al., 2021**)	N/A	BNT162 b2 (2)	DMEK	21	Circumcorneal injection, AC inflammation	Topical dexamethasone	No
**20 papers and 33 patients** (**Kuziez et al., 2023**)	N/A	BNT162 b2 (37%) CoronaV ac (3%), AZD122 2 (19%) mRNA- 1273 (37%) BBIBP- CorV (25%)	PK (n=20), DMEK (n=8), DSAEK (n=7), FLEK (n=1)	Mean 11.2 Median 8.5	N/A	N/A	36% of eyes with rejection resolved, 19% improved, 17% showed nil improvement.
**16 papers and 34 patients** (**Moura-Coelho et al., 2023**)	38.9% of eyes had previous grafts	BNT162 b2 (56%) mRNA- 1273 (24%) AZD122 2 (26%) Sinovac (3%)	PK (n=20), DSAEK (n=7), DMEK (n=6), combined PK and KLAL (n=1) LR- CLAL (n=1)	Median 9	N/A	The most frequently prescribed topical CS was dexamethasone 0.1% (n=13), prednisolone acetate 1% (n=6), betamethasone 0.1% (n=4), and difluprednate 0.05% (n=3)	77.8% resolution 13.9% graft failure and 8.3% partial improvement.
**27 papers 37 patients** (**Huang et al., 2022**)	N/A	BNT162 b2 (71.4%) AZD122 2 (23.8%) Sinovac (4.8%)	PK (n=13) DMEK (n=5) DSAEK (n=3)	Mean 10 Median 7	N/A	N/A	Most common adverse corneal event post COVID- 19 vaccination is corneal graft rejection
**46 patients and 55 eyes between December 2020 and** **July 2022** (**Singh R.B. et al., 2022**)	N/A	BNT162 b2 (74%) mRNA- 1273 (26%)	PK (n=34), DSAEK (n=10), DMEK (n=8) DALK (n=2) CLAT (n=1)	BNT162 b2 mean: 15.1 mRNA- 1273 mean: 19.3	Decreased VA (39%) corneal oedema (20%) AC inflammation (17%) DM folds (17%), conjunctival hyperaemia (15%) KPs (15%)	Topical (n=24) oral (n=4) and subconjunctivally injected (n=5) corticosteroids	37% graft failure Longer vaccination to rejection time in patients with PKs
**7 papers 9 patients** (**Sen and Honavar, 2021**)	N/A	BNT162 b2 (77%) AZD122 2 (33%)	N/A	Mean 7	N/A	N/A	Most common adverse corneal event post COVID- 19 vaccination is corneal graft rejection
**5 corneal papers, 7 patients**	N/A	BNT162 b2 (80%)	PKP (n=4)	N/A	N/A	N/A	Insufficient evidence to suggest causation between COVID-10
(**Wang et al., 2022**)		AZD122 2 (20%)	DMEK (n=3)				vaccination and corneal graft rejection
**4 papers and 6 patients** (**Lee and Huang, 2021**)	N/A	BNT162 b2 (80%) AZD122 2 (20%)	PK (n=2) and DMEK (n=4)	Mean 14	N/A	N/A	Rate of ocular complications following COVID- 19 vaccinations lower than previously reported
**3 corneal papers and 3 patients** (**Kumari et al., 2022**)	N/A	BNT162 b2 and AZD122 2	PK (n=2) DMEK (n=1)	N/A	N/A	N/A	Most common adverse ocular event post COVID-19 vaccination was uveal pathology
**77 cases of graft rejection over 3 years** (**Busin et al., 2022**)	N/A	BNT162 b2, AZD122 2 and mRNA- 1273	PKPs, DSAEKS, DMEKs	N/A	N/A	N/A	Nil statistically significant increase in rejection rates during COVID-19 vaccination period
**11 papers and 15 patients relating to cornea** (**Haseeb et al., 2022**)	N/A	BNT162 b2, CoronaV ac, AZD122 2 mRNA-	PK, DALK, DMEK, DSAEK	Mean 11.8	N/A	N/A	COVID-19 vaccination may be associated with corneal complications
		1273, BBIBP- CorV					
**13 papers and 21 patients**	N/A	BNT162 b2, CoronaV ac, AZD122 2, and mRNA- 1273	PK (n=12), DMEK (n=6), DSAEK (n=4), LR- CLAL (n=1)	Mean 10.4 Median 7	Corneal oedema most common clinical finding (87%)	Most commonly topical steroid (52%) followed by a combination of topical and oral steroid (13%)	Graft failure occurred in 39% of eyes
**77F** (**Balidis et al., 2021**)	N/A	mRNA- 1273 (1)	DMEK	7	Diffuse corneal oedema and AC inflammation	Subconjunctival dexamethasone injections and eye drops of 1 mg/mL dexamethasone and hypertonic every 2 h	No
**74M** (**Shah et al., 2022**)	N/A	mRNA- 1273 (1)	DMEK	7	Corneal oedema and KPs	Topical prednisolone acetate 1% every 2 hours	No
**61F** (**Shah et al., 2022**)	N/A	mRNA- 1273 (2)	PK	14	Corneal stroma oedema, Khodadoust’s line	Topical prednisolone acetate 1% hourly	No
**69F** (**Shah et al., 2022**)	N/A	mRNA- 1273 (2)	DSAEK	14	Corneal oedema	Topical prednisolone acetate 1% and difluprednate 0.05% 6x per day	No
**77M** (**Shah et al., 2022**)	N/A	mRNA- 1273 (2)	PK	7	Corneal oedema, KPs AC inflammation	Topical prednisolone acetate 1% 5x per day	No
**Review** (**Dugan and Mian, 2022**)	N/A	N/A	N/A	N/A	N/A	N/A	Risk factors for vaccination associated rejection include PK, repeat graft, infection, poor ocular surface and young age
**566 episodes of corneal graft rejection from 2018 to 2021** across 2 centres (**Roberts et al., 2022**)	N/A	N/A	N/A	N/A	N/A	N/A	Median rates of rejection before and after initiation of vaccination program were not statistically different
**Review** (**Bizimungu C. et al., 2023**)	N/A	N/A	N/A	N/A	N/A	N/A	Link between vaccine and rejection

Cases arranged by outcome and vaccine subtype.

A total of 44 eyes in 42 patients were included (mean age 56.9 with 43.9% female). Of those 27 eyes had penetrating keratoplasties, 6 eyes had DSAEKs, 7 eyes had DMEKs, 2 had DALKs, one had a combined penetrating keratoplasty and keratolimbal allograft (KLAL), and one had a femtosecond laser-assisted penetrating keratoplasty (FLEK). A large proportion of described cases (47.6%, n=20 patients) occurred in individuals with established risk factors for rejection. These include young patients, bilateral transplants, previous graft rejection and a high level of corneal neo-vascularization (Panda et al., 2007). Fourteen patients had previous rejection episodes (32.6%).

**Figure 2:**
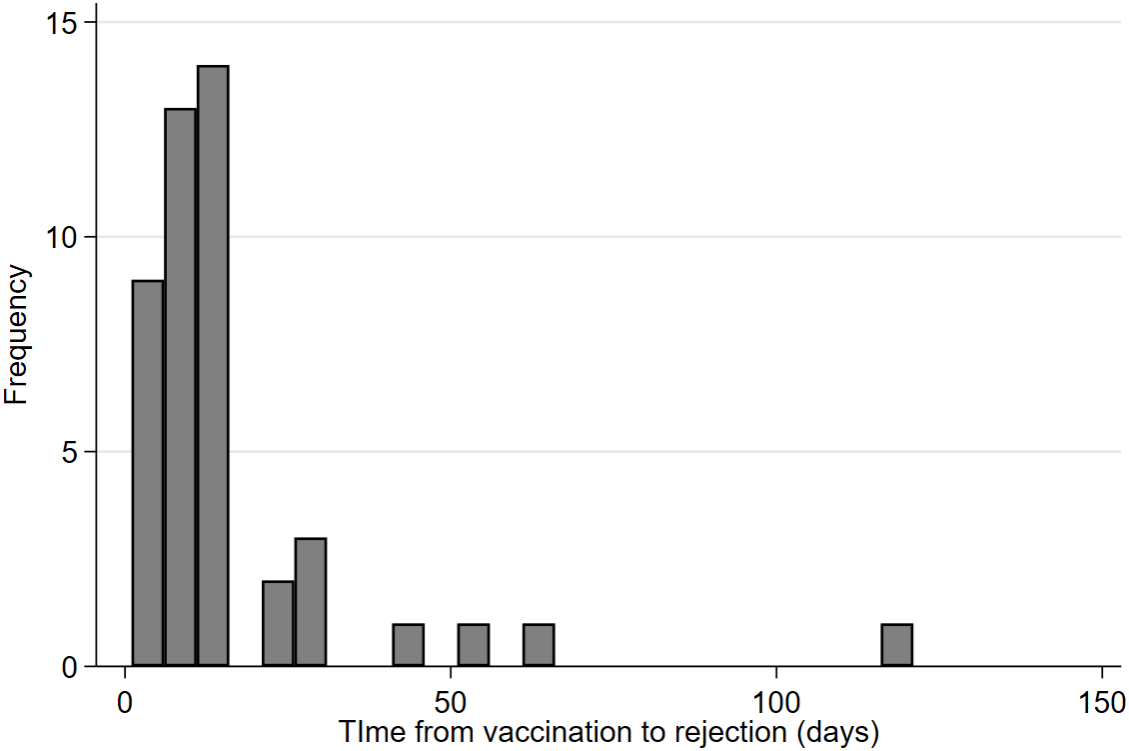
Time from vaccination to rejection episode.

The time between vaccination and rejection episode varied from one day to 117 days post-surgery [**Figure 2**] with a mean of 16.1 days and a median of 11 days [IQR 7 – 14]. No difference was observed in time to rejection by gender (p=0.928) but there was a trend towards faster time to rejection reported in older subjects (p=0.069) and a shorter time from vaccination to rejection was observed in those with risk factors for graft rejection (p=0.030). No difference was observed in the reported time to rejection following vaccination by vaccine type (p=0.5714) or by graft type (p=0.7969). Rejection episodes were treated through a variety of means, mostly topical steroids such as prednisolone or dexamethasone. In 26.2% of cases (n=11) patients were also treated with systemic steroids. Most (79.6%, N=35 eyes) acute rejection episodes resolved with treatment.

The majority of the published literature on COVID-19 vaccination and corneal graft rejection are case reports and small case series. There are no corneal graft and COVID-19 vaccination-specific meta-analyses published to date, but there is a large meta-analysis on organ rejection as a whole showing corneal graft rejection is the most widely reported type of tissue rejection following COVID-19 vaccination in the literature (n=38), accounting for 67.8% of all “organ” rejections (Alhumaid et al., 2022). There are distinct differences between the pathogenesis of (solid organ) rejection and tissue rejection. Solid organ rejection is cell and antibody-mediated whilst tissue rejection is purely cell-mediated, however, the prevalence of both in relation to COVID-19 vaccination suggests an underlying alteration to systemic immunogenicity (Streilein, 2003; Yin, 2021).

Several systematic reviews specific to corneal allograft rejection have been published. The rate of corneal allograft rejection secondary to COVID-19 vaccination is estimated at 0.004 per million, calculated by dividing the number of events by the number of doses (Moura-Coelho et al., 2023). Despite the inferred relation between the vaccine and rejection due to temporal association, within the systematic reviews which discussed corneal allograft rejection following COVID-19 vaccination, none found sufficient evidence to definitively conclude causation (Lee and Huang, 2021; Fujio et al., 2022; Huang et al., 2022; Kumari et al., 2022; Wang et al., 2022; Kuziez et al., 2023; Moura-Coelho et al., 2023). There is significantly more published literature on COVID-19 vaccination and corneal allograft rejection than any other type of vaccination, but the systematic reviews deduce this is likely confounded by the profound public interest in the COVID-19 vaccination, following the global pandemic, encouraging publication (Kuziez et al., 2023; Moura-Coelho et al., 2023).

A large retrospective multi-center study of 471 cases of corneal graft rejection between January 2018 and March 2022 found no difference between rates of corneal graft rejection before COVID-19 and after the introduction of the COVID-19 vaccination (Roberts et al., 2022). This involved a retrospective review of presentations with corneal graft rejection over the study period. The numbers of rejections after the introduction of the COVID-19 vaccination program were compared with the numbers before vaccination and also prior to the pandemic. There was no attempt to link individual graft rejection episodes to the COVID-19 vaccination per se. Similarly, an Italian study of 77 cases of rejection amongst 2062 patients who underwent corneal transplants from January 2018 to December 2021 found no increase in rejection rates following the vaccine scheme implementation in 2021 (Busin et al., 2022). This paper utilized both gross corneal graft rejection numbers and linked the timing of vaccination to a rejection episode, having only one case of rejection out of 77 which occurred within 60 days of receiving a COVID-19 vaccination.

## Discussion

High quality data on the side effects of COVID-19 vaccination is essential as new variants continue to develop and more individuals are immunized (Barouch, 2022). The current literature linking COVID-19 vaccination to corneal transplant rejection is weak, with numbers too small for statistical significance or definitive conclusions. The vast majority of reported literature is individual case reports or case series. Within the reported cases, the intense scrutiny surrounding the vaccine has likely raised suspicion of causation rather than correlation. It is very challenging to ascertain whether a corneal allograft rejection is directly secondary to the COVID-19 vaccination. With around half of cases reported already at baseline high risk of rejection, it is likely some cases were destined for rejection with or without the vaccination. Some papers reported cases as sequelae of the COVID-19 vaccine when the rejection was as long as 117 days post-immunization (Ghiasian et al., 2023). There is no consensus on a sensible interval between vaccine and rejection, and all of these cases were included in our analysis. Further research may benefit from an exclusion criteria of greater than 60 days.

The three larger retrospective studies showed no statistically significant increase in corneal graft rejection with the introduction of the COVID-19 vaccination However, data collection challenges during the pandemic period leave these open for significant confounding issues. During the height of the pandemic, all of the countries involved in these studies experienced some form of lockdown, likely hindering presentation to health services. These studies were all retrospective and relied on accurate clinical coding for their diagnosis of corneal graft rejection which may result in incomplete data. The clinical demographics of the individuals were also not discussed, which is suboptimal as the pre-existing risk factors of a patient play a profound role in the integrity of their immune privilege and hence their likelihood to reject (Streilein, 2003; Maharana et al., 2023).

**Figure 3:**
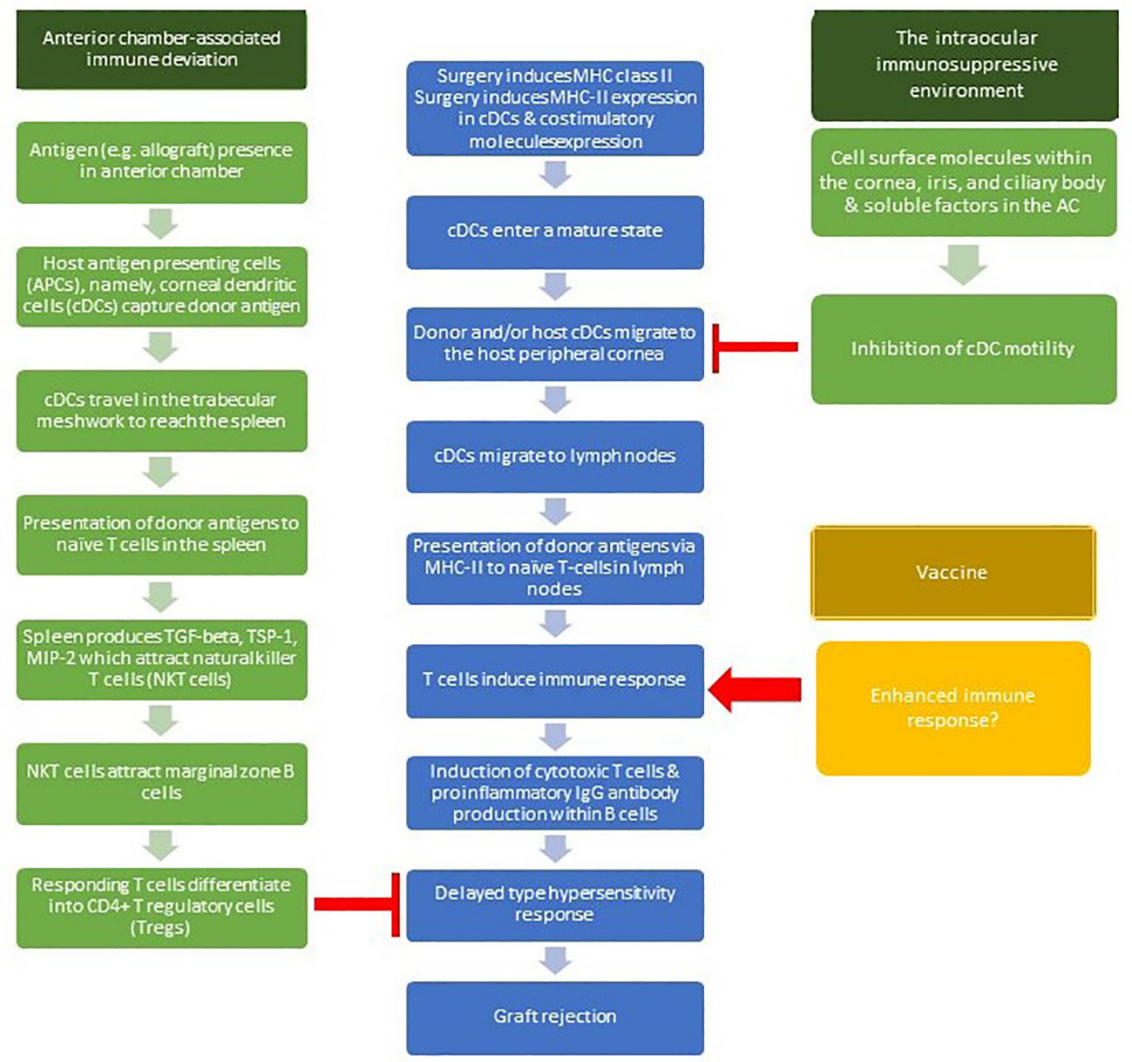
Immunology of corneal graft rejection and the potential impact of vaccination. AC, anterior chamber; APC, antigen presenting cells; cDC, corneal dendritic cells; MIP-2, macrophage inflammatory protein 2; MHC-II, major histocompatibility comlex class II; TGF-beta, transforming growth factor-beta; TSP-1, thrombospondin 1.

The likelihood of developing a rejection following COVID-19 vaccination appears to be correlated to a patient’s underlying risk factors. In 47.3% of cases, patients had established risk factors for rejection and 32.2% had previous rejection episodes. This is likely a result of loss of the immune privilege of the cornea in any circumstance outside of normal physiological conditions, including trauma, inflammation, neovascularization and infection (Niederkorn, 2007). Hence almost all patients undergoing corneal transplantation have compromise of their corneal immune privilege and the potential for rejection. The mechanism for this is widely believed to be CD4+ T cell-mediated via an afferent and effector arm (Niederkorn, 2006; Maharana et al., 2023), demonstrated in **Figure 3**. The afferent branch refers to the presentation of a donor antigen by host APCs. This causes changes in corneal dendritic cells which begin to express major histocompatibility complex (MHC)-II and encourage the release of proinflammatory cytokines (Yamagami et al., 2005). The effector arm refers to T cell activation and allo-reaction in lymph nodes. These sensitized T cells migrate to the cornea with CD4+ T cells recruiting additional leukocytes which mediate tissue damage and form memory T cells, enhancing the immune response seen in repeat corneal graft rejections (Niederkorn, 2007; Hori et al., 2019; Maharana et al., 2023; Yamagami et al., 2005).

The exact mechanism by which vaccination may induce corneal graft rejection is still unclear. COVID-19 vaccination is linked to other ocular pathologies such as uveitis flares (Jordan et al., 2023). Corneal graft rejection has been reported secondary to Hepatitis B, yellow fever, tetanus and influenza vaccinations but the published literature is limited to a handful of case reports (Steinemann et al., 1988; Wertheim et al., 2006; Vignapiano et al., 2022). A proposed mechanism for this association between vaccination and corneal graft rejection is that vaccination increases corneal MHC class II complex antigen expression (Steinemann et al., 1988). The systemic immune response which occurs upon receiving a vaccination may be sufficient inflammation to hinder the normal physiological immune privilege of the cornea (Maharana et al., 2023). Interestingly, our results show that there was a shorter time course between COVID-19 vaccination and rejection in older patients, which contradicts the usual increased rejection risk in younger patients due to their stronger immune response. However, although the data is not available for all these cases, it is more likely that older patients may have had more grafts or graft rejection episodes than younger patients, but there may be additional factors involved beyond the compromise of immune privilege.

The mechanism of corneal transplant rejection is likely dependent on the type of vaccination. The most common causative vaccine in the literature was BNT162b2 (Pfizer) at 33.3% of cases. However, notably, Pfizer was also the most widely used vaccine in this time period (Barouch, 2022). Most vaccines contain an attenuated or inactivated virus or bacteria, while mRNA vaccines introduce mRNA that translates into a protein on a virus’s outer membrane (Dugan and Mian, 2022). BNT162b2 (Pfizer) is a nucleoside modified mRNA vaccine that elicits a strong adaptive humoral and cellular immune response. In large scale randomized control trials participants showed raised levels of CD4+ T helper cells and inflammatory cytokines such as interferon-gamma, as well as CD8+ T cells by day 29 post-vaccination (Anderson et al., 2020; Sahin et al., 2021). These immune factors have all been implicated in the pathogenesis of corneal graft rejection. It was also suggested that there was cross-reactivity between the SARS-CoV-2 antigen and MHC-antigen complexes present in the cornea. Therefore, when an mRNA vaccine introduces a corresponding SARS-CoV-2 mRNA, this may activate and upregulate MHC-antigen complexes, thereby inducing inflammatory processes and rejection (Rajagopal and Priyanka, 2022; Kuziez et al., 2023). There is significant cross-reactivity documented between mRNA vaccines and COVID-19 variant strains suggesting non-specific antigen binding which may extend to the cornea (Melo-González et al., 2021). An analysis of the adverse events global database revealed more vaccine associated rejection events following the second dose of COVID-19 vaccination, suggesting that established antigenic sensitization leads to a larger immunological response. This analysis also noted no reported corneal graft rejection episodes following Adenovirus vector based vaccines as compared to mRNA vaccines (Singh et al., 2022).

There is yet to be a statistically significant study published documenting any vaccination definitively provoking corneal graft rejection. As corneal graft rejection is an uncommon event, part of this discrepancy may be associated with the large sample size required to ascertain a significant association, as well as changes in health-seeking behavior at the height of the COVID pandemic, which may further confound the interpretation of trends. Despite the lack of confirmed association, there is a significant perceived association both clinically and in the published literature (Lee and Huang, 2021; Lockington, 2021; Lockington et al., 2021; Singh et al., 2022; Moura-Coelho et al., 2023). It may be prudent to discuss the option of prophylaxis before vaccination, particularly in patients with known risk factors for rejection, or in “only eyes”. This must be balanced with the associated risks of topical steroid treatment including microbial keratitis and raised intraocular pressure (Kim et al., 2016). Demonstrating successful mitigation of risk through increased topical steroid use will avoid contributing to vaccine hesitancy and reassure patients that a corneal graft is not a contraindication to receiving a vaccination (Lockington, 2021). Health literacy around the clinical signs of rejection which patients may observe should remain a priority for ensuring prompt presentation and treatment.

## Limitations

The primary limitation of this review is the scarcity of large-scale series, with the majority of published data correlating COVID-19 vaccination and corneal graft rejection being isolated case reports or small series. This meant there were some inconsistencies with the way data was reported or made available. Where possible, case series were included and compared with other systematic reviews. The relatively small number of original papers meant that the same data was referenced repeatedly across several review articles and systematic reviews. However, we only included original case reports/series in our analysis and excluded data which was reproduced in other reports. Moreover, there may have been papers missed in the search criteria, should the titles or abstracts not contain the keywords. However, this review provides a thorough overview of COVID-19 vaccination-associated corneal graft rejection and the possible immunological mechanisms for this. The data to date suggests strong correlation between vaccination and allograft rejection in corneal transplantation however, the evidence is not yet convincing of cause and effect. Nonetheless, since billions of COVID-19 vaccinations have been administered it is likely that in the future that more concrete data may be available and therefore more definitive conclusions in regard to the aetiology of corneal graft rejection in the post pandemic population may be defined. Whilst correlation does not necessarily imply causality in terms of COVID-19 vaccination and the rejection episode, the clear reporting of associated comorbidities and risk factors allows for these to be taken into consideration in the analysis.

## Conclusion

The relationship between COVID-19 vaccination and corneal graft rejection is complex and fraught with confounding factors. It appears unlikely that a significant degree of causative relationship will be established in the literature in the near future, and keratoplasty is not a contraindication for vaccination. Early recognition of symptoms and signs of rejection by both patients and clinicians is essential to prevent vision loss and graft failure. Clinician discretion and patient concerns with respect to vaccination are required when considering topical steroid prophylaxis in high-risk corneal graft patients. Further investigation into the mechanism of vaccination-associated corneal graft rejection may provide additional insight in the future.

## Author contributions

NA: Writing – original draft. JZ: Writing – review & editing. CM: Methodology, Supervision, Writing – review & editing.
